# State Minimum Wage and Food Insecurity Among US Households With Children

**DOI:** 10.1001/jamanetworkopen.2025.2043

**Published:** 2025-03-27

**Authors:** Megan R. Winkler, Rachel Clohan, Kelli A. Komro, Melvin D. Livingston, Sara Markowitz

**Affiliations:** 1 Department of Behavioral, Social, and Health Education Sciences, Emory University, Rollins School of Public Health, Atlanta, Georgia; 2 Department of Economics, Emory University, Atlanta, Georgia

## Abstract

**Importance:**

Food insecurity among households with children and economic hardship is a persistent US challenge. Federal food assistance programs have been unable to fully address food insecurity, leading to interest in the role other labor and economic policies could play. One relevant state-level policy that has received limited attention is the state minimum wage.

**Objective:**

To assess whether state minimum wage generosity was associated with change in food insecurity among households with children and explore differential policy impacts across sociodemographic groups.

**Design, Setting, and Participants:**

This cross-sectional study of a national sample of US households from the Current Population Survey used a 2-way fixed effects modeling approach to test whether increases in state minimum wage from 2005 to 2022 were associated with improvements in food insecurity controlling for household- and state-level time-varying covariates. Working households with children who were most likely to be affected by policy changes (ie, limited educational attainment) were included. Analyses were conducted in July through September 2024.

**Exposure:**

The 2022 inflation-adjusted effective minimum wage for each state in December which was derived from legal sources.

**Main Outcomes and Measures:**

Past-month household food insecurity assessed in December 2022.

**Results:**

The sample of 97 944 working households with children and limited educational attainment were mostly female headed (54 077 [55.2%]) with a mean (SD) 1.8 (1.1) children in the home; 22 130 households (22.6%) reported Hispanic identity, 10 545 non-Hispanic Black (10.8%), and 59 500 non-Hispanic White (60.8%). Inflation-adjusted state minimum wage ranged from $7.15 to $16.85 over the 18-year study period. We observed that a 10% increase in the state minimum wage was significantly associated with a 0.39 percentage point reduction (95% CI, −0.74 to −0.04 percentage points; *P* = .03) in food insecurity. There was limited evidence of differences in the association across race and ethnicity, participation in the US Supplemental Nutrition Assistance Program, or household composition.

**Conclusions and Relevance:**

In this pooled cross-sectional study, findings suggest that state legislatures that elected to increase their state minimum wage may have also improved state food security rates among households with children at risk for economic hardship. Findings provide policymakers with actionable evidence to consider in setting minimum wages that could reduce the burden of food insecurity among US children and families.

## Key Points

**Question:**

Are increases in the US state minimum wage associated with reduced food insecurity among economically at-risk households with children?

**Findings:**

In this pooled cross-sectional study using repeated surveys from 2005 to 2022 from a nationally representative sample of US households, more generous state minimum wage policies were associated with significant reductions in household food insecurity. At the federal minimum wage, the estimated level of food insecurity was 9.9%, whereas at the highest state minimum wage the estimated rate was 6.8%.

**Meaning:**

This study suggests that more generous state minimum wage policies may reduce food insecurity among households with children at risk for economic hardship.

## Teaser

This cross-sectional study of national survey data from US households examines the association of state minimum wage with household food insecurity.

## Introduction

Food insecurity among households with children is a persistent challenge in the US. For 2 decades, food insecurity rates have remained above 12% among households with children and rose to 17.3% in 2022.^1^ While disproportionately burdening households with limited economic resources,^2^ rates have also been found to vary across US states, with households in some states experiencing 3 times the prevalence of food insecurity than those in other states.^1^ The consequences of experiencing childhood food insecurity are significant and far-reaching, affecting physical health, child development, academic performance, and mental health.^3,4^

The US Supplemental Nutrition Assistance Program (SNAP) is the largest federal food assistance program designed to address food security challenges. Yet, critical gaps remain in SNAP benefits keeping pace with rising food costs.^5^ These gaps were especially salient during the pandemic, when 50% to 65% of households with children reported using their emergency child tax credit provisions to cover food expenses.^6^ As a result, there is growing interest among clinicians, researchers, and advocates in the role other economic and labor policies—ones unspecific to food—could play in bolstering food security among US children.^7,8,9,10,11^

One relevant policy that has been limitedly examined is state minimum wage. State minimum wage laws act in tandem with the federal policy, which establishes the floor for US workers’ hourly wages. The federal minimum wage was set at $7.25 per hour in 2009 and has not been increased since. Without increases, the real value of minimum wage declines over time due to rises in the cost-of-living, such as food, leading several state legislatures to upwardly amend their state minimum wage for workers. This has led to wide variation in the generosity of the minimum wage across states, with 10 states in 2021 implementing a minimum of at least $12 per hour and 16 states maintaining a minimum wage at or less than the federal.^12^

Differing from economic policies that target households with children, like the child tax credit,^7,8,9^ minimum wage is a labor policy that affects any household with a worker making at or near minimum wage. In fact, state minimum wage increases have been shown to ripple throughout the low-wage workforce up to workers with wages in the 25th percentile.^13^ Among workers who would benefit from minimum wage increases, nearly 30% are estimated to be parents,^14^ and such changes could be especially beneficial for single-parent working households where 42% make low wages.^15^ Moreover, this policy change could address some of the key concerns identified with SNAP, which has been critiqued for distributing benefits on a monthly cycle and, in some states, requires a high applicant burden to become and remain enrolled. In contrast, increases in low-wage family incomes due to state minimum wage changes would be automatic and potentially more consistent occurring on a weekly-to-biweekly basis. While higher minimum wages can enhance incomes among those working, there is a possibility for disemployment changes if the rate is set above the competitive level; yet, the latest empirical work finds these changes to be limited.^16^

Despite the potential state minimum wage increases may have for reducing food insecurity among households with children, US research evaluating these impacts is sparse. Evaluations looking at changes in nutrition from rises in local or state minimum wage have largely been limited to adult populations.^17,18,19^ Only one 2017 study by Bartfeld and Men^11^ was identified as examining state minimum wage impacts on food insecurity among households with children, which found inconsistent results. However, their analysis of households across different levels of education and income examined the relationship between annual food insecurity—or overall experiences between January and December—and state minimum wage in December. Such exposure-outcome timing poorly aligns with changes in state minimum wage that occur from mid-to-late in the year, as respondents would be reporting on their food security both before and after the change. Furthermore, their study period, which ended in 2014, precedes the years where the largest variation in state minimum wage has been observed.

Therefore, the objective of this study was to assess whether changes in US state minimum wage generosity associated with changes in food security among households with children during the period of 2005 to 2022. We focused on working households with limited educational attainment and children who are most likely to benefit from a more generous state minimum wage. We also used a measure of food insecurity (past 30 days) to ensure the outcome assessment follows the state minimum wage policy change. Finally, as policy impacts may not be universal, we explored whether there were any heterogeneous changes in food insecurity across the household’s participation in SNAP, self-reported race and ethnicity, and composition (ie, number of children and single- vs 2-parent household).

## Methods

We used household-level 2005-2022 data from the Food Security Supplement of the Current Population Survey (CPS-FSS).^20^ The supplement is administered every December by the US Census Bureau and collects detailed information on food security status from household units across all 50 states. Our sample included households with at least 1 child aged 18 years or younger. We restricted our sample to households with children where the highest educational attainment was an associate degree or less to approximate an intent-to-treat analysis. Food insecurity rates,^1^ median weekly earnings,^21^ and the proportion in low-wage work (ie, making less than $15 per hour)^22^ are similar among those with a high school degree, some college, or associate degree. Moreover, the largest proportions of workers making minimum wage are those reporting a high school degree (36%) or some college or associate degree (37%).^23^ We also restricted our sample to currently working households (97 944 households; see eFigure in Supplement 1). Changes in the state minimum wage will most directly affect households with a member in the workforce, and prior work has found that beneficial changes from wage-setting policies on adult food insecurity only occurred among those who were employed.^24^

This study used publicly available data and was determined by Emory University institutional review board to not be human participants research. Our study meets the Strengthening the Reporting of Observational Studies in Epidemiology (STROBE) reporting guideline for cross-sectional studies.

### Measures

#### State Minimum Wage

**Figure 1.**
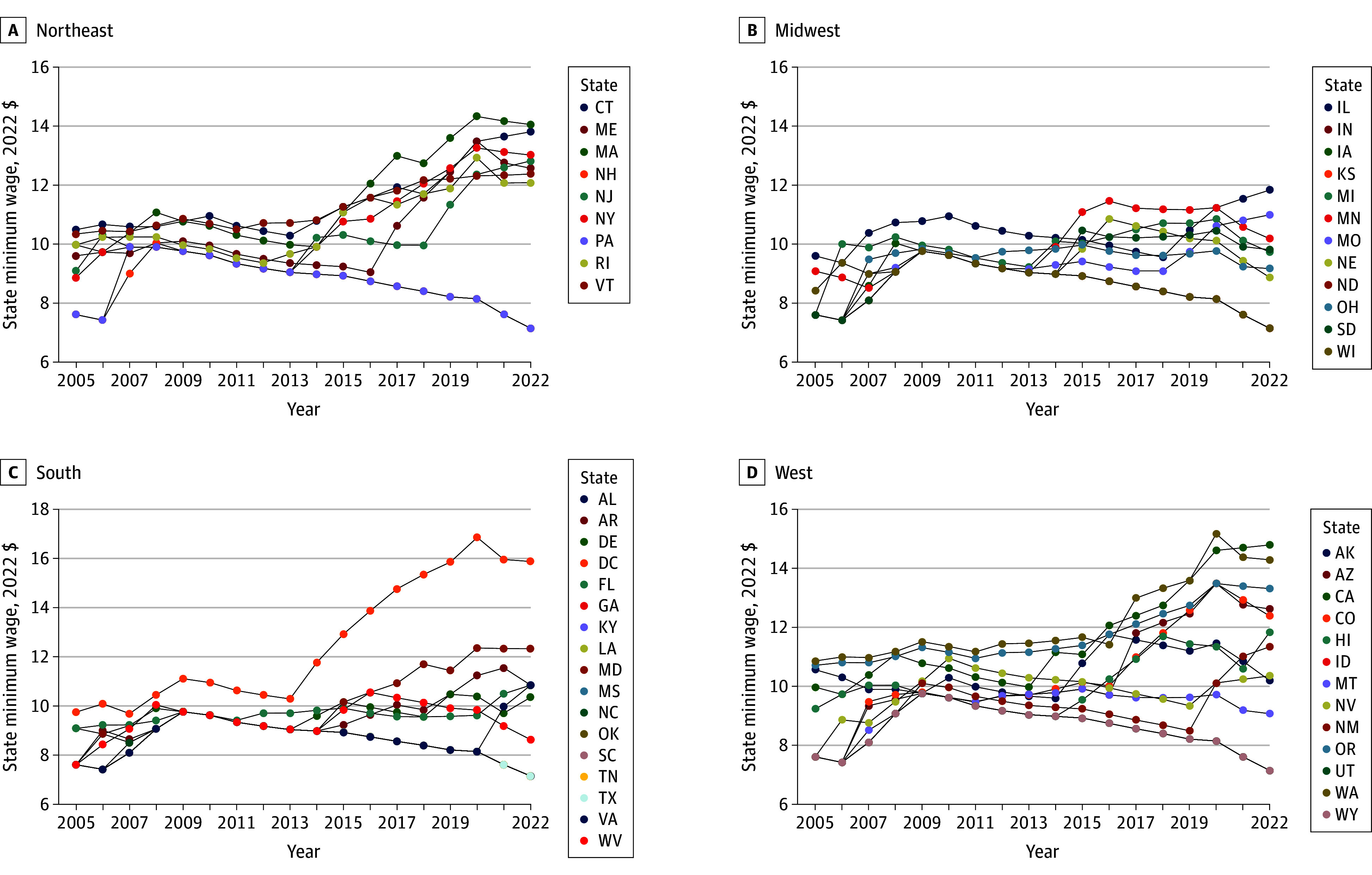
State Minimum Wage Across 50 States by US Census Region, 2005-2022 Values are inflation adjusted to 2022 real US dollars.

State minimum wage was derived from legal sources. The codebook and data from 2005-2020 were collected in collaboration with legal scholars.^25,26^ Data for 2021-2022 followed the same codebook and was collected from state legal sources using masked independent coding by 2 extractors. All divergences were resolved through team discussion and review of legal texts. We used the logged 2022 inflation-adjusted effective minimum wage for each state at the end of the year (December). In states and years where there was not a state minimum wage or it was lower than the federal, the federal minimum was used. Figure 1 displays the change in the effective inflation-adjusted minimum wage across all 50 states over the study period (2005 to 2022). Inflation-adjusted state minimum wage ranged from $7.15 to $16.85 over the 18-year period and 18 states had a minimum wage above the federal level in 2005 compared with 31 states in 2022.

#### Outcome

We measured household food insecurity via the past 30-day measure developed by the USDA^27^ and collected every December since 2005 as part of CPS-FSS. The 18-item measure identifies different levels of food security (secure, marginal, low, or very low) in the past 30 days.^28^ We selected the 30-day vs the annual measure to ensure appropriate timing with state minimum wage changes which can occur across the calendar year. The 30-day measure is also likely to capture households experiencing more consistent challenges with food security, as the annual measure captures households with both regular and varying experiences of food insecurity and is a reason annual rates are higher than the past 30-day rates.^11^ We compared households who reported experiencing very low, low, and either low or very low food security (hereafter referred to as food insecurity) to food secure households defined as households with high or marginal food security (ie, 0-2 affirmative responses on the 18-item scale).^28^

#### Covariates

We controlled for several household characteristics relevant to the study question, including the self-reported age, sex, and race and ethnicity of the head of household, the household partner status (single vs married or cohabitating), the highest education level of the household (associate, some college, high school), the number of children living in the household, and whether the household received benefits from the SNAP program and/or the Special Supplemental Nutrition Program for Women, Infants, and Children (WIC). Information on how CPS handles item nonresponse on these covariates is available elsewhere.^29^ To control for state-specific time-varying confounding, we included the following state covariates obtained from the University of Kentucky Center for Poverty Research, including state unemployment rate, real income per capita, percentage of residents experiencing poverty, and the combined monthly maximum benefit for a 3-person family for Temporary Assistance for Needy Families and SNAP programs. We also included state participation in Medicaid expansion and the generosity of state Earned Income Tax Credit.

### Statistical Analysis

We used a 2-way fixed effects approach to examine whether there were improvements in food insecurity rates among working households with children who experienced an increase in their state minimum wage. The approach examines within-state variation over time by estimating the average difference within states before and after the policy after subtracting the differences in states without a policy change (ie, the comparison or control group). The general estimation equation is as follows:

Y_ist_ = β_1_ ln(MW)_st_ + β_2_ X_ist_ + β_3_S_st_ + γ_s_ + τ_t_ + ε_ist_

*Y* is food insecurity outcome for household *i* in state *s* and in December of year *t*. The MW_st_ represents the state minimum wages expressed as a continuous logged 2022-inflation adjusted variable. The model includes the vectors of household characteristics (X_ist_) and time-varying state characteristics (S_st_), state fixed effects (γ_s_), and year fixed effects (τ_t_). The state fixed effects account for unmeasured state-level covariates that are static over time, while year fixed effects account for time-varying covariates common to all states. All models were estimated as linear probability models and standard errors were clustered on state. In main models, we did not include sampling weights as these are developed for the entire CPS sample and cannot be reestimated for our intent-to-treat population of currently working households with children and limited education. As recommended by Solon, Haider, and Woolridge,^30^ we control for the CPS sampling frame in our covariates, including age, race, sex, and state. We estimated separate models for different educational attainment levels (at most associate degree, at most some college, at most high school degree, and less than high school degree) and food security outcomes (very low food security, low food security, and food insecurity).

In addition, we conducted a series of sensitivity analyses and robustness tests (eMethods in Supplement 1). First, we estimated a falsification test among households with a master’s degree or higher to ensure the association was absent in these highly educated households who should be the least impacted by minimum wage policy changes.^23^ Second, we examined how estimates changed when we removed the COVID pandemic years (2020-2022) that were affected by food security safety net expansions, added survey weights, and dropped the working household restriction. We also reestimated our linear probability models as ordered logits, which showed no substantive differences; these are not discussed further.

Finally, we explored whether differential associations existed across household SNAP participation, race and ethnicity (Hispanic, non-Hispanic Black, non-Hispanic White), the number of children living in the home (1, 2, or 3 or more), and the household partner status (single vs married or partnered). We ran stratified models for each group and due to the limited sample size, we report both 95% and 90% confidence intervals. We used the 95% CIs to determine statistical significance for this exploratory analysis of differential associations across different social and demographic groups (SNAP, race and ethnicity, number of children, and household partner status). All analyses were conducted using the software package of Stata SE, version 17.0 (StataCorp).

## Results

The sample of 97 944 working households with children and at most an associate degree was mostly female headed (54 077 [55.2%]) with a mean (SD) 1.8 (1.1) children in the home; 22 130 households (22.6%) reported Hispanic identity, 10 545 non-Hispanic Black (10.8%), and 59 500 non-Hispanic White (60.8%) (Table 1). Table 2 shows the results for state minimum wage increases on food insecurity outcomes among the limited education groups. Similar reductions in overall food insecurity were observed for working households with children who have at most an associate degree, some college, or a high school education. For households with at most an associate degree, a 10% increase in minimum wage was associated with a reduction of 0.39 percentage points (95% CI, −0.74 to −0.04 percentage points) in the food insecurity rate—which translates to a 3.4% reduction or approximately 58 401 fewer households with food insecurity per year nationally. Accordingly, the estimated level of food insecurity among households with at most an associate degree at the federal minimum wage was 9.9%, whereas at the highest state minimum wage value the estimated rate was 6.8% (eTable 1 in Supplement 1). Changes were largely attributed to households experiencing low vs very low food security (Table 2). For households with less than a high school education, there were no significant changes in food security rates following minimum wage changes (Table 2). As hypothesized, there was also not an association between minimum wage and food insecurity among households with advanced education (eTable 2 in Supplement 1). Results and conclusions also remained relatively unchanged across the different sensitivity analyses, including when the COVID pandemic years (2020-2022) were removed (eTables 3 through 5 in Supplement 1).

**Table 1.** Sample Characteristics Among Working Households With Children and Limited Education, 2005-2022

Characteristics	Households, No. (%)^a^			
	At most associate degree (n = 97 944)	At most some college (n = 85 154)	At most high school degree (n = 44 669)	Less than high school degree (n = 10 188)
Past-month outcome				
Food secure	86 787 (88.6)	74 987 (88.1)	38 720 (86.7)	8423 (82.7)
Food insecurity (low or very low)	11 157 (11.4)	10 167 (11.9)	5949 (13.3)	1765 (17.3)
Low food security	7855 (8.0)	7169 (8.4)	4205 (9.4)	1265 (12.4)
Very low food security	3302 (3.4)	2998 (3.5)	1744 (3.9)	500 (4.9)
Household characteristics				
Age, mean (SD), y	38.3 (8.9)	38.1 (9.0)	38.0 (9.2)	38.3 (9.2)
Sex of household head				
Male-headed household	43 867 (44.8)	38 043 (44.7)	19 985 (44.7)	4465 (43.8)
Female-headed household	54 077 (55.2)	47 111 (55.3)	24 684 (55.3)	5723 (56.2)
Household structure				
Single-parent household	33 806 (34.5)	30 289 (35.6)	16 852 (37.7)	3822 (37.5)
Married or partnered	64 138 (65.5)	54 865 (64.4)	27 817 (62.3)	6366 (62.5)
Race and ethnicity				
Hispanic	22 130 (22.6)	20 642 (24.2)	14 945 (33.5)	6723 (66.0)
Non-Hispanic Asian or Pacific Islander	3033 (3.1)	2623 (3.1)	1494 (3.3)	305 (3.0)
Non-Hispanic Black	10 545 (10.8)	9433 (11.1)	4885 (10.9)	814 (8.0)
Non-Hispanic Native American	1272 (1.3)	1097 (1.3)	541 (1.2)	83 (0.8)
Non-Hispanic multiple racial identities	1464 (1.5)	1235 (1.5)	525 (1.2)	60 (0.6)
Non-Hispanic White	59 500 (60.8)	50 124 (58.9)	22 279 (49.9)	2203 (21.6)
No. of children in the home, mean (SD)	1.8 (1.1)	1.8 (1.1)	1.9 (1.1)	2.1 (1.2)
WIC benefit recipiency				
Received	8982 (9.2)	8344 (9.8)	5342 (12.0)	1742 (17.1)
Not Received	45 375 (46.3)	40 607 (47.7)	22 976 (51.4)	5890 (57.8)
Missing	43 587 (44.5)	36 203 (42.5)	16 351 (36.6)	2556 (25.1)
SNAP benefit recipiency				
Received	15 946 (16.3)	14 772 (17.4)	9386 (21.0)	2854 (28.0)
Not received	43 190 (44.1)	38 463 (45.2)	21 407 (47.9)	5385 (52.9)
Missing	38 808 (39.6)	31 919 (37.5)	13 876 (31.1)	1949 (19.1)
State-level covariates				
Unemployment rate, mean (SD)	6.0 (2.3)	6.0 (2.3)	6.1 (2.3)	6.3 (2.3)
Real income per capita, in thousands, mean (SD), $	56.9 (9.2)	56.8 (9.2)	57.0 (9.4)	57.7 (9.5)
Poverty rate, mean (SD)	13.0 (3.2)	13.0 (3.2)	13.2 (3.2)	13.6 (3.1)
Combined monthly maximum for TANF and SNAP (3-person family), mean (SD), $	936.3 (198.9)	933.3 (199.1)	930.9 (200.9)	937.7 (202.4)
Medicaid expansion				
Lives in state without	75 790 (77.4)	66 243 (77.8)	34 684 (77.7)	7839 (76.9)
Lives in state with	22 154 (22.6)	18 911 (22.2)	9985 (22.4)	2349 (23.1)
State earned income tax credit policy in the state				
No or nonrefundable tax credit	64 708 (66.1)	56 468 (66.3)	29 775 (66.7)	6797 (66.7)
Tertile 1 (lowest credit values)	13 123 (13.4)	11 415 (13.4)	5670 (12.7)	1266 (12.4)
Tertile 2	9666 (9.9)	8327 (9.8)	4322 (9.7)	858 (8.4)
Tertile 3 (highest credit values)	10 447 (10.7)	8944 (10.5)	4902 (11.0)	1267 (12.4)

Abbreviations: SNAP, US Supplemental Nutrition Assistance Program; TANF, Temporary Assistance for Needy Families; WIC, Special Supplemental Nutrition Program for Women, Infants, and Children.

^a^ Measures for each characteristic include numbers and proportions in the analytic sample unless otherwise specified in the row label for the characteristic.

**Table 2.** Past-Month Food Insecurity Among Working Households With Children and Limited Education, 2005-2022^a^

Result	Coefficient (95% CI)		
	Food Insecurity^b^	Low Food Security^c^	Very Low Food Security^d^
**At most associate degree**			
10% increase in state minimum wage	−0.39 (−0.74 to −0.04)	−0.27 (−0.54 to −0.01)	−0.17 (−0.39 to 0.05)
*P* value	.03	.04	.13
Overall mean	0.114	0.083	0.037
No.	97 944	94 642	90 089
**At most some college**			
10% increase in state minimum wage	−0.38 (−0.75 to −0.01)	−0.26 (−0.53 to 0.01)	−0.17 (−0.42 to 0.09)
*P* value	.04	.06	.18
Overall mean	0.119	0.087	0.038
No.	85 154	82 156	77 985
**At most high school degree**			
10% increase in state minimum wage	−0.39 (−0.85 to 0.08)	−0.29 (−0.65 to 0.08)	−0.16 (−0.50 to 0.19)
*P* value	.09	.12	.37
Overall mean	0.133	0.098	0.043
No.	44 669	42 925	40 464
**Less than high school degree**			
10% increase in state minimum wage	0.11 (−0.98 to 1.19)	−0.21 (−1.18 to 0.76)	0.37 (−0.31 to 1.06)
*P* value	.84	.66	.28
Overall mean	0.173	0.131	0.056
No.	10 188	9688	8923

^a^ Robust standard errors clustered by states. All models are among working households and control for self-reported age, sex, race and ethnicity of the head of household; household marital or partner status; the number of children living in the household; household Special Supplemental Nutrition Program for Women, Infants, and Children and/or Supplemental Nutrition Assistance Program (SNAP) benefit recipiency; state unemployment rate; state real income per capita; state percentage of poverty; state participation in Medicaid expansion; the combined state monthly maximum benefit for Temporary Assistance for Needy Families and SNAP for a 3-person family; state Earned Income Tax Credit; as well as state and year effects. In addition to the regular controls, associate degree results control for high school education and some college, and some college results control for high school education. The state minimum wage is the logged 2022 inflation-adjusted effective minimum wage for each state at the end of the year. All coefficients have been multiplied by 10 to facilitate interpretation. A 0.39 percentage point reduction in the food insecurity rate among the at most associate degree analytic sample approximates 58 401 households who are no longer food insecure per year nationally.

^b^ Models compare households who were identified as low or very low food security (food insecure households) to those who were identified as food secure.

^c^ Models compare households who were identified as low food security to those who were identified as food secure. It excludes households with very low food security.

^d^ Models compare households who were identified as very low food security to those who were identified as food secure. It excludes households with low food security.

**Figure 2.**
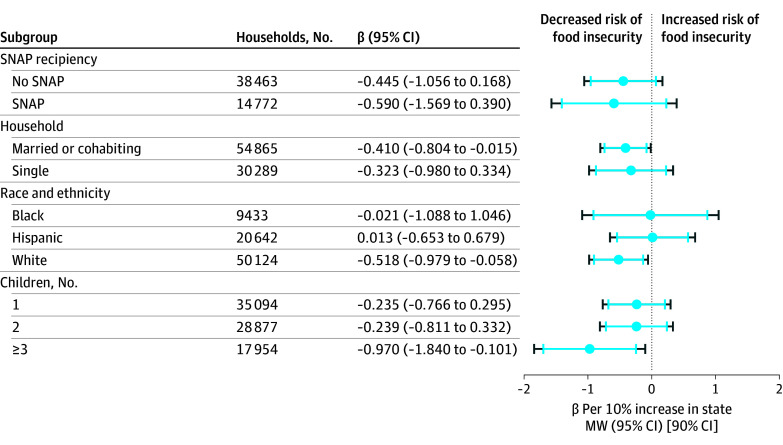
Effects of State Minimum Wage on Past-Month Food Insecurity Across Subgroups, 2005-2022 Estimated effect of the natural log of state minimum wage on food insecurity rates. Models are stratified by each characteristic and use robust standard errors clustered by states. All models are among working households with at most some college education (85 154 households total). Models control for self-reported age, sex, race and ethnicity of the head of household; household educational attainment; household marital or partner status; the number of children living in the household; household Special Supplemental Nutrition Program for Women, Infants, and Children and/or Supplemental Nutrition Assistance Program (SNAP) benefit recipiency; state unemployment rate; state real income per capita; state percentage of residents experiencing poverty; state participation in Medicaid expansion; the combined state monthly maximum benefit for Temporary Assistance for Needy Families and SNAP for a 3-person family; state Earned Income Tax Credit; as well as state and year effects. Black and blue lines represent 95% and 90% confidence intervals (CIs), respectively. The state minimum wage is the logged 2022-inflation adjusted effective minimum wage for each state at the end of the year. All coefficients have been multiplied by 10 to facilitate interpretation. We did not perform analyses on subgroups with <3000 household observations, which included non-Hispanic Asian American or Pacific Islander, non-Hispanic Native American, and Non-Hispanic multiple racial identities.

Figure 2 shows the associations between increases in state minimum wage and food insecurity across different sociodemographic groups. The associations that occurred in the overall sample were consistent for most subgroups and reached statistical significance for partnered households (β, −0.410; 95% CI, −0.804 to −0.015) and households with 3 or more children (β, −0.970; 95% CI, −1.840 to −0.101). There was also some indication that the policy may be most helpful for non-Hispanic White households (β, −0.518; 95% CI, −0.979 to −0.058) compared with Hispanic (β, 0.013; 95% CI, −0.653 to 0.679) and non-Hispanic Black households (β, −0.021; 95% CI, −1.088 to 1.046).

## Discussion

Using a 2-way fixed effects design, we find increases in state minimum wages were associated with improvements in food insecurity among households with children and at risk of economic hardship. This association was largely consistent across different cut-offs of limited educational attainment, persisted across different model specifications and assumptions, and absent in our falsification test among highly educated households. We also observed minimal heterogeneity in the association across subgroups, although we found that partnered households, households with 3 or more children, and non-Hispanic White households especially benefited from increases in state minimum wages.

Our results suggest meaningful short-run impacts on food insecurity among economically challenged households with children. Such improvements in food insecurity rates due to rises in the state minimum wage are important, as child consequences of food insecurity are far-reaching^3,4^ and research indicates that even minor increases in food security can translate to improvements in health.^31^ Similar beneficial effects from local minimum wage increases have been found for the number of calories purchased per adult among households making the minimum wage or less.^32^ These authors also found some support that the healthfulness of purchases improved the most for those with the least healthy purchasing patterns before the policy change,^32^ suggesting that households may not only acquire additional foods but potentially more nutritious options.

We also found that the beneficial changes from increases in state minimum wage were similar across a variety of social groups and household contexts. This advances on the scant prior research examining nutritional outcomes of state minimum wage by uncovering who may benefit the most from these changes. Despite all groups having overlapping confidence intervals and most similar point estimates, we found there may be especially salient changes for married and partnered households and for households with multiple children. While we did not formally test whether there were different associations across race and ethnicity, we found no association among Hispanic and non-Hispanic Black households and a significant beneficial change among non-Hispanic White households. Similar patterns across racial groups have been found for other health and well-being challenges,^33,34,35^ highlighting that groups who experience additional forms of social disadvantage can face diminished returns from their socioeconomic gains.^36^ At the same time, given the disproportionate exposure Hispanic and non-Hispanic Black workers have to working in low-wage positions,^37^ it is possible that the average reductions in food insecurity could still translate to important declines for these groups. Either way, more substantive economic and labor policies are necessary to overcome the known financial gaps in incomes across racial and ethnic groups, which persist even across limited education.^38^

### Strengths and Limitations

Strengths of our study included the quasi-experimental design with an intention-to-treat analysis and falsification tests, examining recent years when the generosity of state minimum wage reached its greatest divergence, and controlling for a host of individual- and state-level confounders. Furthermore, as inaccuracies in policies have been identified in data from administrative sources, our study notably advances on prior work by using a minimum wage policy dataset that was generated following best practices in legal epidemiology.^39^

An important limitation of our study was the use of the CPS data itself to identify food insecure households. To limit participant burden, CPS only asks the 18-item measure for households that are below 185% of the poverty line and positively respond to 1 of 2 preliminary screening questions—assuming all others are food secure. Therefore, some food insecure households may be assumed to be food secure through this screening procedure. Furthermore, we used the CPS developed measure of food insecurity, which combines marginal food secure households with food secure households. While less severe than experiences of low food security, marginal food security has shown relevant impacts on child outcomes, especially younger aged children,^4^ and may have resulted in an underestimation of change in food security.

## Conclusions

In this pooled cross-sectional study of households with children, we estimated significant improvements in food security that were associated with increases in the state minimum wage. This study adds to the growing body of research on the potentially beneficial health effects observed for children and families through improved labor and economic support policies.^40^ Limited movement at the federal level and increasing variation in state policy choices is progressively leading to a patchwork of supports for families depending on where they live. Therefore, these study results have implications for both state and federal policymakers and suggest that minimum wage increases could be a useful strategy to help reduce US childhood food insecurity.

## Notes

Supplement 1. **eFigure.** Flow Diagram of Analytic Samples Generated From the Current Population Survey Food Security Supplement (2005-2022)
eMethods.
**eTable 1.** Estimated Past Month Food Insecurity Among Working Households With Children and Limited Education (2005-2022) **eTable 2.** Past Month Food Insecurity Among Working Households With Children Where the Highest Educational Attainment Is a Master’s Degree or Higher (2005-2022) **eTable 3**. Past Month Food Insecurity Among Working Households With Children and Limited Education, Excluding COVID Pandemic Years (2005-2019) **eTable 4**. Past Month Food Insecurity Among Working Households With Children and Limited Education With Sampling Weights (2005-2022) **eTable 5**. Past Month Food Insecurity Among All Households (Unrestricted to Current Work Status) With Children and Limited Education (2005-2022)
eReferences.

Supplement 2.
Data Sharing Statement
