# Supplementary material for: State Minimum Wage and Food Insecurity Among US Households With Children
Source: JAMA Netw Open. 2025 Mar 27;8(3):e252043. doi: 10.1001/jamanetworkopen.2025.2043 (PMC11950886; doi:10.1001/jamanetworkopen.2025.2043)
Supplement: Supplement 1. — eFigure. Flow Diagram of Analytic Samples Generated From the Current Population Survey Food Security Supplement (2005-2022) eMethods. eTable 1. Estimated Past Month Food Insecurity Among Working Households With Children and Limited Education (2005-2022) eTable 2. Past Month Food Insecurity Among Working Households With Children Where the Highest Educational Attainment Is a Master’s Degree or Higher (2005-2022) eTable 3. Past Month Food Insecurity Among Working Households With Children and Limited Education, Excluding COVID Pandemic Years (2005-2019) eTable 4. Past Month Food Insecurity Among Working Households With Children and Limited Education With Sampling Weights (2005-2022) eTable 5. Past Month Food Insecurity Among All Households (Unrestricted to Current Work Status) With Children and Limited Education (2005-2022) eReferences. [file jamanetwopen-e252043-s001.pdf]

Supplemental Online Content

Winkler MR, Clohan R, Komro KA, Livingston MD, Markowitz S. State minimum wage and food insecurity among US households with children. *JAMA Netw Open.* 2025;8(3):e252043. doi:10.1001/jamanetworkopen.2025.2043

eFigure 1. Flow Diagram of Analytic Samples Generated From the Current Population Survey Food Security Supplement (2005-2022)

eTable 1. Estimated Past Month Food Insecurity Among Working Households With Children and Limited Education (2005-2022)

eTable 2. Past Month Food Insecurity Among Working Households With Children Where the Highest Educational Attainment Is a Master's Degree or Higher (2005-2022)

eTable 3. Past Month Food Insecurity Among Working Households With Children and Limited Education, Excluding COVID Pandemic Years (2005-2019)

eTable 4. Past Month Food Insecurity Among Working Households With Children and Limited Education With Sampling Weights (2005-2022)

eTable 5. Past Month Food Insecurity Among All Households (Unrestricted to Current Work Status) With Children and Limited Education (2005-2022)

eReferences.

This supplemental material has been provided by the authors to give readers additional information about their work.

© 2025 Winkler MR et al. *JAMA Network Open*.

eFigure 1. Flow diagram of the analytic samples generated from the Current Population Survey Food Security Supplement (2005-2022).

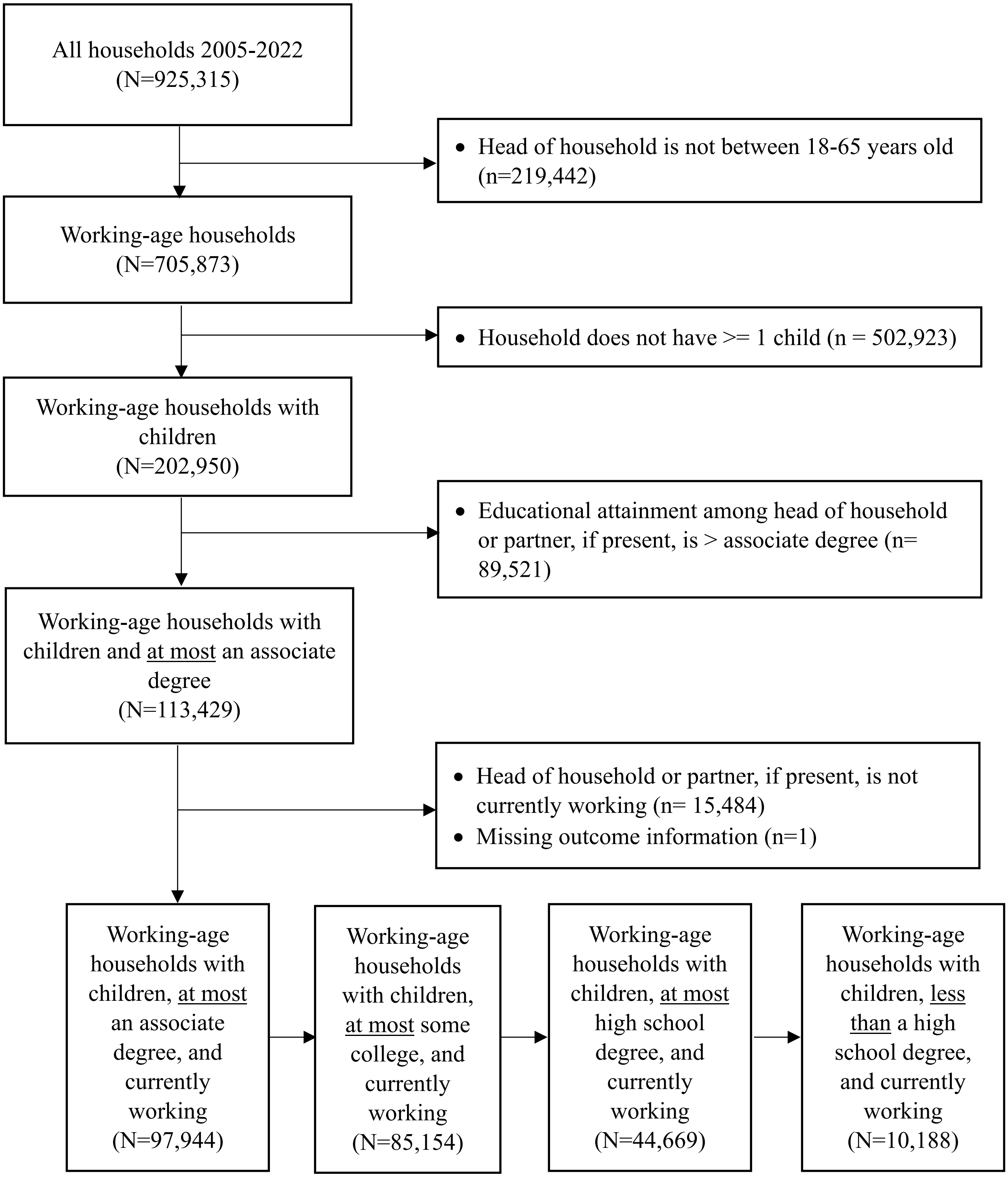

© 2025 Winkler MR et al. *JAMA Network Open*.

eMethods.

1. **Estimated Food Insecurity Rates**

In eTable 1, we report the estimated mean food insecurity rates at the federal minimum wage ln($7.25) and maximum state minimum wage of ln($16.10) (observed in DC in 2020) to facilitate interpretation of the estimates reported in **Table 2.**

2. **Falsification Test**

As a falsification test, we estimated past 30-day food insecurity (low and very low) among working households with children where the highest educational attainment is a master's degree or higher. We selected this education attainment as 13.1% of the minimum wage workforce[1] reports an educational attainment of a Bachelor's degree, whereas only 2.2% report a Master's degree or more—making this group the least likely to be affected by minimum wage policy changes. Further, the proportion of food insecure households reporting a college degree or more has been on the rise (18% in 2017 to 23% in 2022)[2] indicating a Bachelor's education may no longer be as protective of economic hardship as previously considered. As anticipated, results in **eTable 2** indicate no association between changes in state minimum wage and change in food insecurity rates.

3. **Sensitivity Analyses**

COVID Pandemic. The social safety net for food insecurity (i.e., SNAP benefits, increase in summer meals, and charitable donations) changed rapidly during the COVID pandemic years of 2020-2022, as economically vulnerable households were first laid off, then provided an increased safety net, which was subsequently removed in 2022. Food insecurity rates among households with children mapped onto these changes, as rates rose to 14.8% in 2020, dropped to 12.5% in 2021, and rose again to 17.3% in 2022—making the change from

© 2025 Winkler MR et al. *JAMA Network Open*.

2021 to 2022 the second largest annual increase since CPS began tracking household food insecurity[2]. At the same time, state minimum wage showed the widest variability in generosity since 2005 across these 2020-2022 years (see **Figure 1**).

As a sensitivity analysis, we ran models excluding the COVID pandemic years for working households with children and limited educational attainment. We found similar conclusions to when those years were included in the main analysis (**eTable 3**).

Sampling Weights. We did not use sampling weights in our primary models as weights were developed for the entire Current Population Survey Food Security Supplement sample and cannot be re-estimated for our intent-to-treat population of currently working households with children and different levels of limited education attainment. As recommended by Solon, Haider & Woolridge (2015),[3] we control for the CPS sampling frame in our covariates, including age, race, sex, and state, for the main model. As they also recommend, we report estimates including the sampling weights below. Conclusions remain similar despite the decrease in the magnitude of the point estimates and widening of the confidence intervals (**eTable 4**).

Working Household Restriction. Last, we examined policy effect estimates among all households regardless of current working status. We find similar conclusions to our main models with a larger magnitude of the decline in food insecurity (**eTable 5**).

© 2025 Winkler MR et al. *JAMA Network Open*.

eTable 1. Estimated past month food insecurity among working households with children and limited education (2005-2022).

	At Most Associate Degree	At Most Some College	At Most High School Degree	Less than High School Degree
	Estimated Mean			
Estimated food insecurity				
At federal minimum wage	0.099	0.111	0.124	0.127
At maximum state minimum wage	0.068	0.081	0.093	0.136

Note. Estimates are from models among working households using robust standard errors clustered by states. Estimates were obtained by running the margins command in Stata SE version 17.0. Models controlled for age, sex, race/ ethnicity of the head of household; household marital/ partner status; household educational attainment; the number of children living in the household; household WIC and/or SNAP benefit recipiency; state unemployment rate; state real income per capita; state percent poverty; state participation in Medicaid expansion; the combined state monthly maximum benefit for TANF and SNAP for 3-person family; state Earned Income Tax Credit; as well as state and year effects. Food insecurity rates were estimated at federal minimum wage of ln($7.25) and maximum state minimum wage of ln($16.10) (observed in DC in 2020).

© 2025 Winkler MR et al. *JAMA Network Open*.

eTable 2. Past month food insecurity among working households with children where the highest educational attainment is a master's degree or higher (2005-2022).

	Coefficient (95% CI)
10% increase in state minimum wage	-0.04<br>(-0.21,0.12)
Observations	37905
Overall Mean	0.027

Note. CI= confidence interval. Robust standard errors clustered by states. The model is among working households where the highest educational attainment among the head of household or partner (if present) is a Master's degree or higher. The model controls for age, sex, race/ ethnicity of the head of household; household marital/ partner status; the number of children living in the household; household WIC and/or SNAP benefit recipiency; state unemployment rate; state real income per capita; state percent poverty; state participation in Medicaid expansion; the combined state monthly maximum for TANF and SNAP for 3-person family; state Earned Income Tax Credit; as well as state and year effects. The state minimum wage is the logged 2022-inflation adjusted effective minimum wage for each state at the end of the year. All coefficients have been multiplied by 10 to facilitate interpretation.
$^{*}p < 0.10$, $^{**}p < 0.05$, $^{***}p < 0.01$

© 2025 Winkler MR et al. *JAMA Network Open*.

eTable 3. Past month food insecurity among working households with children and limited education, excluding COVID pandemic years (2005-2019)[a]

	Food Insecurity[b]	Low Food Security[c]	Very Low Food Security[d]
	Coefficient (95% CI)		
Panel A At Most Associate Degree			
10% increase in state minimum wage	-0.38* (-0.79, 0.04)	-0.27* (-0.59, 0.05)	-0.16 (-0.43,0.10)
Overall mean	0.116	0.084	0.037
N	87942	84915	80786
Panel B At Most Some College			
10% increase in state minimum wage	-0.41* (-0.85, 0.02)	-0.25 (-0.60,0.10)	-0.22 (-0.50,0.06)
Overall mean	0.121	0.089	0.039
N	76706	73955	70149
Panel C At Most High School Degree			
10% increase in state minimum wage	-0.55* (-1.16,0.05)	-0.32 (-0.84,0.20)	-0.33* (-0.72,0.06)
Overall mean	0.135	0.099	0.044
N	40163	38572	36334
Panel D Less than High School Degree			
10% increase in state minimum wage	-0.29 (-1.79,1.21)	-0.47 (-1.78,0.84)	0.18 (-0.73,1.09)
Overall mean	0.174	0.131	0.056
N	9266	8814	8110

[a] Robust standard errors clustered by states. All models are among working households and control for age, sex, race/ ethnicity of the head of household; household marital/ partner status; the number of children living in the household; household WIC and/or SNAP benefit recipiency; state unemployment rate; state real income per capita; state percent poverty; state participation in Medicaid expansion; the combined state monthly maximum benefit for TANF and SNAP for 3-person family; state Earned Income Tax Credit; as well as state and year effects. The highest educational attainment among the head of household or partner (if present) is an Associate Degree (Panel A), Some College (Panel B), or High School Degree (Panel C). In addition to the regular controls, Panel A controls high-school education and some college, and Panel B controls for high-school education. The state minimum wage is the logged 2022-inflation adjusted effective minimum wage for each state at the end of the year. All coefficients have been multiplied by 10 to facilitate interpretation.

[b] Models compare household who were identified as low or very low food security (food insecure households) to those were identified as food secure.

[c] Models compare households who were identified as low food security to those who were identified as food secure. It excludes households with very low food security.

[d] Models compare households who were identified as very low food security to those who were identified as food secure. It excludes households with low food security.

* $p < 0.10$, ** $p < 0.05$, *** $p < 0.01$

© 2025 Winkler MR et al. *JAMA Network Open*.

eTable 4. Past month food insecurity among working households with children and limited education with sampling weights (2005-2022) [a]

	Food Insecurity[b]	Low Food Security[c]	Very Low Food Security[d]
	Coefficient (95% CI)		
Panel A At Most Associate Degree			
10% increase in state minimum wage	-0.30<br>(-0.67, 0.07)	-0.20<br>(-0.47, 0.06)	-0.14<br>(-0.38,0.10)
Overall mean	0.114	0.083	0.037
N	97944	94642	90089
Panel B At Most Some College			
10% increase in state minimum wage	-0.29<br>(-0.69, 0.10)	-0.21<br>(-0.47,0.06)	-0.13<br>(-0.42,0.17)
Overall mean	0.119	0.087	0.038
N	85154	82156	77985
Panel C At Most High School Degree			
10% increase in state minimum wage	-0.18<br>(-0.62,0.27)	-0.15<br>(-0.53,0.23)	-0.05<br>(-0.44,0.34)
Overall mean	0.133	0.098	0.043
N	44669	42925	40464
Panel D Less than High School Degree			
10% increase in state minimum wage	0.26<br>(-0.62,1.15)	-0.18<br>(-0.97,0.60)	0.58<br>(-0.13,1.30)
Overall mean	0.173	0.131	0.056
N	10188	9688	8923

[a] Robust standard errors clustered by states. All models are among working households and control for age, sex, race/ ethnicity of the head of household; household marital/ partner status; the number of children living in the household; household WIC and/or SNAP benefit recipiency; state unemployment rate; state real income per capita; state percent poverty; state participation in Medicaid expansion; the combined state monthly maximum benefit for TANF and SNAP for 3-person family; state Earned Income Tax Credit; as well as state and year effects. The highest educational attainment among the head of household or partner (if present) is an Associate Degree (Panel A), Some College (Panel B), or High School Degree (Panel C). In addition to the regular controls, Panel A controls high-school education and some college, and Panel B controls for high-school education. CPS weights are included. The state minimum wage is the logged 2022-inflation adjusted effective minimum wage for each state at the end of the year. All coefficients have been multiplied by 10 to facilitate interpretation.

[b] Models compare household who were identified as low or very low food security (food insecure households) to those were identified as food secure.

[c] Models compare households who were identified as low food security to those who were identified as food secure. It excludes households with very low food security.

[d] Models compare households who were identified as very low food security to those who were identified as food secure. It excludes households with low food security.

* $p < 0.10$, ** $p < 0.05$, *** $p < 0.01$

© 2025 Winkler MR et al. *JAMA Network Open*.

eTable 5. Past month food insecurity among all households (unrestricted to current work status) with children and limited education (2005-2022) [a]

	Food Insecurity[b]	Low Food Security[c]	Very Low Food Security[d]
	Coefficient (95% CI)		
Panel A At Most Associate Degree			
10% increase in state minimum wage	-0.44** (-0.79, -0.09)	-0.32** (-0.61, -0.03)	-0.20* (-0.41,0.02)
Overall mean	0.135	0.096	0.047
N	113426	108544	103002
Panel B At Most Some College			
10% increase in state minimum wage	-0.43** (-0.80, -0.06)	-0.32** (-0.63, -0.01)	-0.19 (-0.42,0.04)
Overall mean	0.142	0.101	0.050
N	99597	95105	89986
Panel C At Most High School Degree			
10% increase in state minimum wage	-0.41* (-0.86,0.04)	-0.29 (-0.69,0.10)	-0.20 (-0.50,0.10)
Overall mean	0.159	0.114	0.056
N	54242	51510	48364
Panel D At Most Less than High School Degree			
10% increase in state minimum wage	-0.09 (-1.15,0.97)	-0.23 (-1.19,0.73)	0.10 (-0.53,0.73)
Overall mean	0.207	0.152	0.076
N	13698	12801	11756

[a] Robust standard errors (SEs) clustered by states in parentheses. All models are among working households and control for age, sex, race/ ethnicity of the head of household; household marital/ partner status; the number of children living in the household; household WIC and/or SNAP benefit recipiency; state unemployment rate; state real income per capita; state percent poverty; state participation in Medicaid expansion; the combined state monthly maximum for TANF and SNAP for 3-person family; state Earned Income Tax Credit; as well as state and year effects. The highest educational attainment among the head of household or partner (if present) is an Associate Degree (Panel A), Some College (Panel B), or High School Degree (Panel C). In addition to the regular controls, Panel A controls high-school education and some college and Panel B controls for high-school education. The state minimum wage is the logged 2022-inflation adjusted effective minimum wage for each state at the end of the year. All coefficients have been multiplied by 10 to facilitate interpretation.

[b] Models compare household who were identified as low or very low food security (food insecure households) to those were identified as food secure.

[c] Models compare households who were identified as low food security to those who were identified as food secure. It excludes households with very low food security.

[d] Models compare households who were identified as very low food security to those who were identified as food secure. It excludes households with low food security.

* $p < 0.10$, ** $p < 0.05$, *** $p < 0.01$

© 2025 Winkler MR et al. *JAMA Network Open*.

eReferences

1. Characteristics of minimum wage workers, 2022. Bureau of Labor Statistics. Accessed August 27, 2024. https://www.bls.gov/opub/reports/minimum-wage/2022/

2. USDA ERS - Interactive Charts and Highlights. Accessed August 27, 2024. https://www.ers.usda.gov/topics/food-nutrition-assistance/food-security-in-the-u-s/interactive-charts-and-highlights/#disability

3. Solon G, Haider SJ, Wooldridge JM. What Are We Weighting For? *The Journal of Human Resources*. 2015;50(2):301-316.

© 2025 Winkler MR et al. *JAMA Network Open*.
