# Supplementary material for: State Minimum Wage and Food Insecurity Among US Households With Children
Source: JAMA Netw Open. 2025 Mar 27;8(3):e252043. doi: 10.1001/jamanetworkopen.2025.2043 (PMC11950886; doi:10.1001/jamanetworkopen.2025.2043)
Supplement: Supplement 2. — Data Sharing Statement [file jamanetwopen-e252043-s002.pdf]

Data Sharing Statement

Winkler. State Minimum Wage and Food Insecurity Among US Households With Children. *JAMA Netw Open*. Published March 27, 2025. doi:10.1001/jamanetworkopen.2025.2043

Data
Data available: No

Additional Information
Explanation for why data not available: Data from the Current Population Study is made publicly available from IPUMS. The policy data used and analyzed during the current study are available from the corresponding author on reasonable request.
